# Buried Alive!

**Published:** 1888-05-05

**Authors:** 


### The Hospital

A Weekly Institutional Journal of

Science, Medicine, Nursing, and Philanthropy.

*For Hospitals, Asylums, Medical Practitioners, Students, Nurses, Families, Parishes, Congregations, and the Charitable Public.*

Vol. IV. No. 84.] [May 5, 1888.

### Buried Alive!

The Reverend Harry Jones, who was for many years the well-known rector of St. George's-in-the-East, is now publishing in *Good Words* his Reminiscences, chiefly relating to his personal experiences as a London clergyman. Mr. Jones has always been known for the breadth and strength of his humanity, and for wholesome common sense. He is responsible for the following stories: A certain young woman, within his own knowledge, was attacked with cholera, and was pronounced to be dead. She was "laid out" for the undertaker's collecting cart to call for her on its rounds, when a neighbour happened to come in. "And is poor Sarah really dead?" cried she. "Well," said the mother, "she is, and will soon be fetched away; but if you can do anything you may do it." Thus encouraged, the hopeful neighbour began to rub the supposed dead body energetically with mustard. In no long time Sarah sat up, and so far recovered as to marry; "and I myself," says Mr. Jones, "christened four or five of her children in the next few years."

Mr. Jones relates also the case of a potman, within his own experience, who was similarly supposed to be dead, or so nearly that death was considered certain. A doctor was called in. "Turn him on his face," said the man of physic, "and I will put a thick strip of flannel soaked in spirits of wine down his spine. We will see what that will do." A quantity of flannel was brought, which the doctor soaked in spirit and prepared to apply as he had proposed. First, however, he placed the mass in a heap on the patient's upturned back. A sister, who was assisting, leant forward with a lighted candle in her hand, and accidentally set fire to the soaked flannel as it lay on the back. The pain of the burning roused the potman up, "and," adds Mr. Jones, "not very long ago the doctor told me he had seen him in a street near the Oxford Circus." Arguing from these cases, known as facts to himself, and from other recorded cases, as well as from probability, Mr. Jones comes to the conclusion that in times of panic from fatal epidemics it is not unlikely that some people are buried alive.

That is a sufficiently startling conclusion, and seems to be warranted by the facts. It may perhaps be taken for granted that in certain epidemics, when people are alarmed out of all self-control; when the ordinary instincts of humanity are dominated, and, to a large extent, replaced by the one imperious feeling of mortal terror; when every person attacked and dying is believed to be a powerful source of infection which prudence declares ought to be removed at the earliest possible moment; at such times it perhaps need not be doubted that some people are buried alive. The persons to whom this dreadful fate has happened are not likely to have been the feeble and weak, who on waking up would soon die from mere fright and exhaustion, but the strong and the robust, who although prostrated until every appearance of death was present, yet were not killed outright by the attack, and who might therefore live for some time and realise to the full all the horrors of their situation. It makes the flesh creep and the heart sink to picture such a fate happening to our loved ones or even to ourselves.

We do not call attention to the subject for the purpose of supplying a dish of horrors to those who love such fare; neither, we are sure, did Mr. Harry Jones dig up out of the pit of memory those dreadful stories for any such purpose. Our object is to consider for a moment whether it be scientifically and practically probable that such instances do actually occur with more or less frequency; and whether anything can be done to prevent them. The past we cannot recall; but every one of us who lives in a large town where epidemics must sometimes prevail, is bound to say and do everything he can to prevent such occurrences of dread and terror repeating themselves.

How can a man appear to be dead when he really is not dead? Can he cease to breathe, and his heart to pulsate perceptibly, and yet be alive? Can he deceive the doctor who is trained to know and to speak with authority on all these points? It is to be feared that an affirmative answer must be given to both these questions. The most scientific medical men do not know what life is with sufficient understanding to enable them to define it precisely. All kinds of definitions have been offered, but as these are more or less merely curious, we need not reproduce them here. The fact is, that we do not know exactly what life is. The same is true of death, except that we may define it when applied to a once-living being as "the absence of life."

But though we do not know what life is with that degree of scientific clearness of perception which will enable us to define it with precision and to obtain a unanimous agreement with our definition, we know certain things about it which are sufficient to prevent us going very far wrong. We know, for example, that the breathing and the beating of the heart are essential to normal life; and that if these be suspended entirely for a certain length of time decomposition is bound to set in; and when that has commenced decidedly the patient is dead. But the breathing and the beating of the heart are produced by nerve-energy, and by that alone. Stop the flow of nerve-energy, and you stop the heart's pulsation and the expansion and contraction of the lungs. But is that necessarily death? Certainly not. Although the flow of nerve-energy may be checked absolutely by a sudden shock, vital activity may simply be suspended, not destroyed. It is probably at this very point where the mistakes have been made which have resulted in people being buried alive. Take the case of a strong young man or woman, well-fed, accustomed to physical exercise, suffering from no inherited or acquired taint which has lowered the vitality. Such a one is struck with an unusually severe attack of cholera, whilst all the organs and tissues are in a condition of perfect health. The system receives a very severe shock—not a shock of a momentary character, which may be recovered from as suddenly as it came, after a brief interval; but a slower shock extending over several hours. That slow shock is sufficient to exhaust the nerve-energy of the brain and cord and other ganglia to such an extent as to make it impossible for them to give off enough to maintain the processes of respiration and cardiac pulsation. The patient does not breathe, or does not breathe perceptibly; his heart does not beat, or if it does the ear of the acutest auscultator cannot hear it. Is the patient dead? We cannot tell. How long may he live in that condition? We do not know. For the moment the action of life is gone. But is life gone? It is utterly impossible to say.

What, then, is to be done in such a case as is here imagined? A very simple thing. The patient should be left alone for a time in a well-warmed but thoroughly-ventilated room. On no account should he be put into a coffin and closed up. The other members of the family must avoid panic, and be content to run such risk as there is for a few hours more. After a sufficient length of time—say six, twelve, or twenty-four hours—one or several efforts should be made under the medical attendant's direction to arouse the nervous system to renewed action. A sharp rubbing may be all that is required, as in the case of the young woman who was restored by the friction with mustard; or a prompt and energetic shock may be necessary, such as was successful with the potman whose back was burnt by the blazing spirits of wine. If such mesures were uniformly taken in those instances where the patient seemed previously healthy and very tenacious of life, it is probable that such horrors as Mr. Harry Jones's stories suggest to the imagination would become things of the past. It need not be doubted that cases of the kind are rare; but with all the resources of civilization at our command, they ought to be rendered impossible. Men and women, husbands and wives, fathers and mothers, must bear in mind that much of the responsibility rests with themselves. In epidemic periods, when the doctor is harassed with innumerable calls and is in hourly danger of breaking down himself, he can only direct; he cannot be at every case to carry out thoroughly his own instructions. Heads of households, and all who have reached adult age, should know the dangers and the possible hopes of such cases, and should learn to act for themselves with intelligence, with promptitude, and with vigour.

